# Shear wave elastography of the spleen: evaluation of spleen stiffness in healthy volunteers

**DOI:** 10.1007/s00261-016-0834-4

**Published:** 2016-07-07

**Authors:** Aleksander Pawluś, Marcin S. Inglot, Kinga Szymańska, Krzysztof Kaczorowski, Bartosz D. Markiewicz, Agnieszka Kaczorowska, Jacek Gąsiorowski, Aleksandra Szymczak, Małgorzata Inglot, Joanna Bladowska, Urszula Zaleska-Dorobisz

**Affiliations:** 1 Department of Radiology, Wrocław Medical University, Wrocław, Poland; 2 Department of General and Pediatric Radiology, Independent Public Clinical Hospital Number 1, Ul. M. Curie-Skłodowskiej 68, 50-369 Wrocław, Poland; 3 Medical University of Silesia, Katowice, Poland; 4 Department of Immunology and Pediatrics, J. Gronkowski Provincial Specialist Hospital in Wrocław, Wrocław, Poland; 5 Department of Infectious Diseases, Wroclaw Medical University, Wrocław, Poland

**Keywords:** Spleen, Elastography, SWE, Shear wave elastography

## Abstract

**Purpose:**

The aim of this study was to assess the mean value of spleen stiffness measured by Shear wave elastography in healthy patients and its dependence on age, sex, and spleen dimensions, and to evaluate the repeatability of this method.

**Methods:**

The final study group included 59 healthy volunteers without any clinical evidence of liver disease, portal hypertension, hematological disorders, and without any pathological ultrasonographic spleen findings. Each patient underwent abdominal ultrasound examination and elastography of the liver and the spleen.

**Results:**

The mean value of spleen stiffness was 16.6 ± 2.5 kPa. In the group of men (*N* = 25), it was 17.3 ± 2.7 kPa, and in the group of women (*N* = 34), it was 16.1 ± 2.2 kPa. The study confirmed no correlation between spleen stiffness and sex, age of patients, and spleen size. Coefficient of repeatability and correlation coefficient between the results of the first and the second measurement showed good but not ideal repeatability of the measurement results.

**Conclusion:**

Our outcomes may be a reference point for evaluating spleen stiffness in research on patients with various illnesses.

Elastography is a relatively new diagnostic method used to assess stiffness (elasticity) of soft tissues [1]. Its usefulness is based on the conclusion that pathological changes tend to be harder and less elastic than surrounding healthy tissue. For example, breast or prostate tumors are frequently observed as hard, nodulous changes. What is more, it is generally agreed that no other parameter changes as rapidly in pathologically altered tissue as elasticity [2].

Shear wave elastography (SWE) is, next to transient elastography (TE) or acoustic radiation force impulse (ARFI), one of the most advanced elastography techniques. All of them rely on measuring shear waves propagation and allow to assess tissue stiffness in a quantitative way. Shear waves in TE are induced mechanically by the “thump” at the skin surface, whereas in ARFI technique it is a result of ultrasound-induced radiation force at selected depth [1, 3].

The concept of SWE relies on generating transverse waves by using an acoustic radiation force created by a focused ultrasound beam at different depths. The waves create a new, transverse cone-shaped shear wave. To estimate propagation of the shear wave, an unusually quick data acquisition by the ultrasound (US) probe is required, whereas slower techniques are sufficient in TE and ARFI methods. The SWE device must be able to register at least 5000 frames per second [4]. Due to ultrafast data acquisition, it is possible to make the final estimation result independent of unintentional patient or physician movements. SWE also enables production of a two-dimensional color map of stiffness which is superimposed on the real-time grayscale US image. It allows the visualizing of morphology and measurement of elasticity of the examined area simultaneously [4]. The results of the SWE examination are given in kilopascals (kPa) or in m/s. Elastography plays an important role in investigation of many disease conditions such as liver fibrosis or breast tumors [3].

Single studies of spleen elastography have been published so far. They have shown a potential usefulness of spleen elastography in estimating the risk of esophageal varices in patients with liver cirrhosis [5, 6] or portal hypertension [7].

However, it is necessary to provide information about spleen elasticity in healthy people, without chronic liver diseases, hematological disorders, and viral infections.

The results of two studies on the value of SWE spleen stiffness in healthy volunteers have been published. In 2011, Arda et al. reported mean values of SWE spleen stiffness as 3.1 ± 1.9 kPa among men and ±2.9 kPa among women without a statistically significant difference in elasticity values between sexes [8]. The mean value of spleen elasticity measured by Leung et al. in 2013 on a control group of healthy volunteers was determined as 17.3 ± 2.6 kPa with no statistically significant difference between men and women [9]. As shown above, the differences in these two publications were striking.

The aims of this research were to estimate the average value of SWE spleen stiffness, its correlation with age, sex, spleen size, and to evaluate repeatability of this method, and therefore estimate the groundwork for further investigations concerning spleen sonoelastography.

## Materials and methods

The study was conducted in accordance with the guidelines of the local University Ethics Committee for conducting research involving humans. Each patient signed his/her informed consent before participation in the examination.

**Fig. 1:**
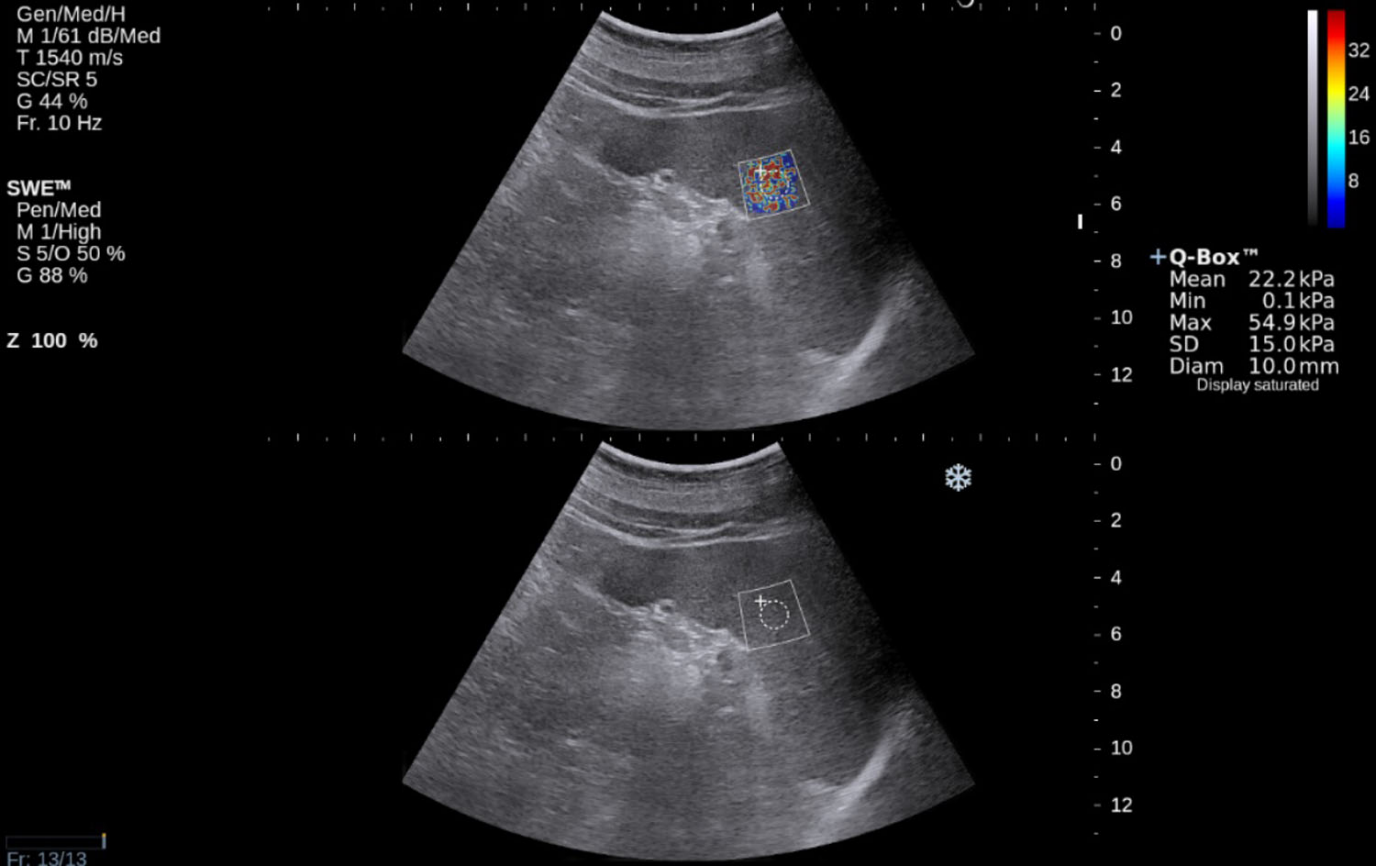
SWE spleen view—measurement unreliable due to too large standard deviation. Probably it is the result of movement artifacts

A group of 70 adult volunteers without any history of chronic liver diseases, portal hypertension, or hematological disorders underwent abdominal US examination and SWE liver elastography. The studied group consisted mainly of the hospital staff, students, or their relatives. Patients with focal splenic lesions, portal vein or bile tract dilatation, significant liver steatosis (evaluated in subjective way according to assessment of liver parenchyma echogenicity itself and in comparison to the right renal cortex), and SWE liver elasticity value higher than 6.5 kPa were excluded from the next examination stage. As a result, 63 volunteers were selected for further research. The examination was performed using an Aixplorer device (Aixplorer Ultrasound System, SuperSonic Imagine SA, France). The ultrasound examination, as well as elastography, was performed by an experienced radiologist. Spleen SWE examinations were performed through intercostal spaces with the patient lying supine with the arms behind his head. In order to minimize breathing motion, the patient was instructed to inhale deeply and hold his breath. This position eliminates artifacts and allows one to visualize the spleen most precisely. All elastographic measurements were performed with a convex transducer (Supersonic Imaging System) with a frequency range 1–6 MHz. Transverse and longitudinal dimensions of the spleen were also measured. It was impossible to measure SWE spleen stiffness in four patients due to significant artifacts as shown in Fig. 1.

**Fig. 2:**
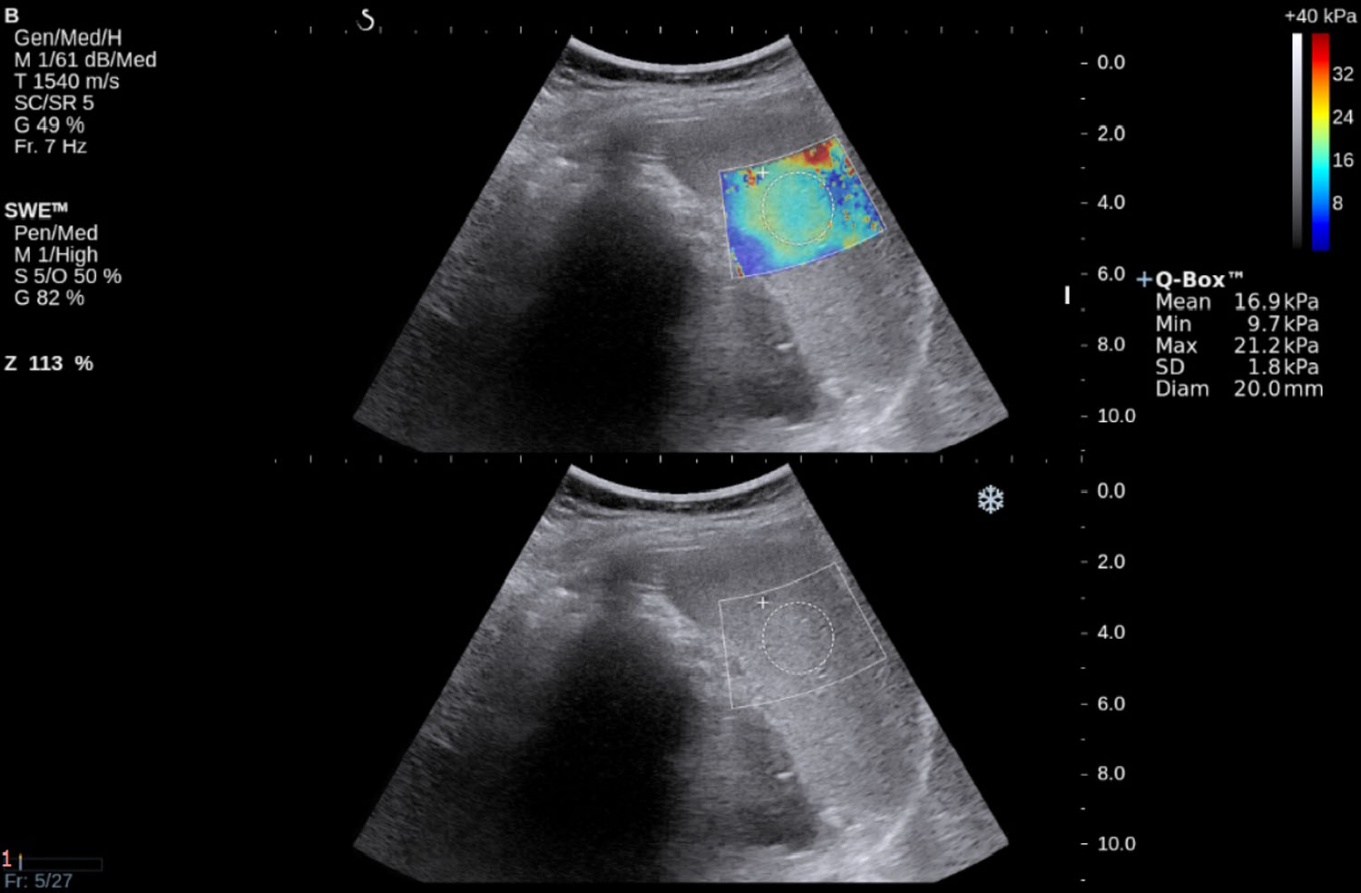
SWE spleen view. ROI put in a place of homogeneous hardness

The final study group included 59 volunteers. For more information, see Table 1. The examinations were performed using dual mode (B-mode and B-mode with a color-coded map of stiffness as an overlay). A region of the central splenic parenchyma with homogeneous elasticity and without the presence of big vessels was selected. The largest possible region of interest (ROI) was placed in such a region and stiffness measurement was recorded as shown in Fig. 2. This procedure was performed twice in each patient in the same area of the spleen.

**Table 1:** Basic data about the examined group

Parameter	Value
Number of participants	59
Number of men*	25 (42)
Number of women*	34 (58)
Age^a^	36 (26) [21–80]
Men’s age^a^	40 (25) [21–80]
Women’s age^a^	34 (22.5) [22–77]
Longitudinal spleen diameter^b^	9.6 ± 1.1
Transverse spleen diameter^b^	4.6 ± 0.9
Body mass index^c^	23.8 [18–30.9]

^a^Median, IQR in parentheses, range in brackets

^b^Average, ±SD

^c^Average, range in brackets

* Percentage ratio in parentheses

To analyze age correlation, subjects were divided in two ways. In the first one, two subgroups were created—patients aged up to 45, *N* = 40; and patients aged 45 and above, *N* = 19. In the second way, volunteers were divided into three subgroups: patients aged up to 34, *N* = 24; patients aged from 35 up to 49, *N* = 17; and above 49, *N* = 18.

The dimension correlation was analyzed after dividing the acquired values of transverse and longitudinal spleen dimensions into two groups based on a median. The median for a longitudinal dimension was 9.6 cm, and for a transverse dimension—4.5 cm.

To verify the normal distribution, the Shapiro–Wilk test was used.

The *U* Mann–Whitney test was used to identify correlations between spleen stiffness and sex as well as spleen size and age in two age subgroups: up to 45 years and above.

To assess correlation between spleen stiffness and age in three subgroups—up to 34 years, from 35 to 49 years, and over 49—the Dunn’s test was applied.

To estimate the repeatability of spleen SWE, three statistical methods were used: the Bland–Altman test, the R&R method, and calculation of correlation coefficient. The confidence intervals on the difference between the result of the first and the second measurement were stated at the 95% confidence level.

All statistical calculations were carried out using statistical computing software Statistica v. 10 (StatSoft, Inc. Tulsa, USA).

## Results

### Spleen stiffness and its dependence on sex

The mean value of SWE spleen stiffness was 16.6 ± 2.5 kPa. In the group of men, it was 17.3 ± 2.7 kPa, and in the group of women, it was 16.1 ± 2.2 kPa. There was no statistically significant difference between these two groups (*p* = 0.102). For more information, see Table 2.

**Table 2:** SWE spleen stiffness and its dependence on sex

	Altogether *N* = 59	Sex		W vs. M
		Women *N* = 34	Men *N* = 25	
Spleen stiffness (kPa)				*p* = 0.102
M ± SD	16.6 ± 2.5	16.1 ± 2.2	17.3 ± 2.7	
Me (*Q*_1_; *Q*_3_)	16.6 (15.0; 18.0)	16.2 (14.7; 17.9)	16.9 (15.8; 18.4)	
Min ÷ Max	10.8–23.1	11.4–21.2	10.8–23.1	

### Correlation between spleen stiffness and age

In the subgroup including patients up to the age of 45, the mean value of a spleen stiffness was 16.5 ± 2.4 kPa, while in patients over 45 it was 16.8 ± 2.6 kPa. No statistically significant difference between these two groups was observed (*p* = 0.820). For more information, see Table 3.

**Table 3:** Spleen stiffness (kPa) depending on patients’ age divided into two groups

	Age (years)		From 45 vs. over 45
	Up to 45 *N* = 40	Older than 45 *N* = 19	
Spleen stiffness (kPa)			*p* = 0.820
M ± SD	16.5 ± 2.4	16.8 ± 2.6	
Me (*Q*_1_; *Q*_3_)	16.9 (14.9; 18.0)	16.3 (15.6; 17.8)	
Min ÷ Max	10.8–21.2	11.9–23.1	

The mean values of spleen stiffness in three subgroups, up to the age of 34, from the age of 34 to 49, and over 50 are as follows: in the first subgroup, it was 16.4 ± 2.3 kPa; in the second subgroup, 16.6 ± 2.6 kPa; and in the third subgroup, 16.9 ± 2.6 kPa. No statistically significant difference between these groups was observed (*p* = 0.943). For more information, see Table 4.

**Table 4:** Spleen stiffness (kPa) depending on patients’ age divided into three groups

	Age (years)			
	Up to 34	From 35 to 49	50 and over	
Spleen stiffness (kPa)	*n* = 24	*n* = 17	*n* = 18	*p* = 0.943
M ± SD	16.4 ± 2.3	16.6 ± 2.6	16.9 ± 2.6	
Me (*Q*_1_; *Q*_3_)	16.9 (14.8; 18.0)	16.6 (15.0; 18.1)	16.4 (15.7; 17.9)	
Min ÷ Max	11.4–21.0	10.8–21.2	11.9–23.1	

### Correlation between spleen stiffness and spleen size

In the group with bipolar spleen dimension up to 9.6 cm (*N* = 31), the mean spleen stiffness was 16.5 ± 2.2 kPa; in the group with bipolar spleen dimension above 9.6 cm (*N* = 28), it was 16 ± 2.7 kPa. There was no statistically significant difference between these groups (*p* = 0.836). For more information, see Table 5.

**Table 5:** SWE spleen stiffness and its dependence on longitudinal spleen diameter

	Longitudinal spleen diameter (cm)		From 9.6 vs. more than 9.6
	Up to 9.6 *N* = 31	More than 9.6 *N* = 28	
Spleen stiffness (kPa)			*p* = 0.836
M ± SD	16.5 ± 2.2	16.7 ± 2.7	
Me (*Q*_1_; *Q*_3_)	16.4 (15.0; 18.0)	16.65 (15.0; 18.1)	
Min ÷ Max	10.8–21.1	11.4–23.1	

In the group with transverse spleen dimension up to 4.5 cm (*N* = 30), the average spleen stiffness was 16.7 ± 2.7 kPa; in the group with transverse spleen dimension above 4.5 cm (*N* = 29), it was 16.5 ± 2.2 kPa. There was no statistically significant difference between these groups (*p* = 0.102). For more information, see Table 6.

**Table 6:** SWE spleen stiffness and its dependence on transverse spleen diameter

	Transverse spleen diameter (cm)		From 4.5 vs. more than 4.5
	Up to 4.5 *N* = 30	More than 4.5 *N* = 19	
Spleen stiffness (kPa)			*p* = 0.596
M ± SD	16.7 ± 2.7	16.5 ± 2.2	
Me (*Q*_1_; *Q*_3_)	16.8 (14.8; 18.0)	16.55 (15.1; 17.4)	
Min ÷ Max	10.8–21.8	11.9–23.1	

### Repeatability of spleen SWE examination

**Fig. 3:**
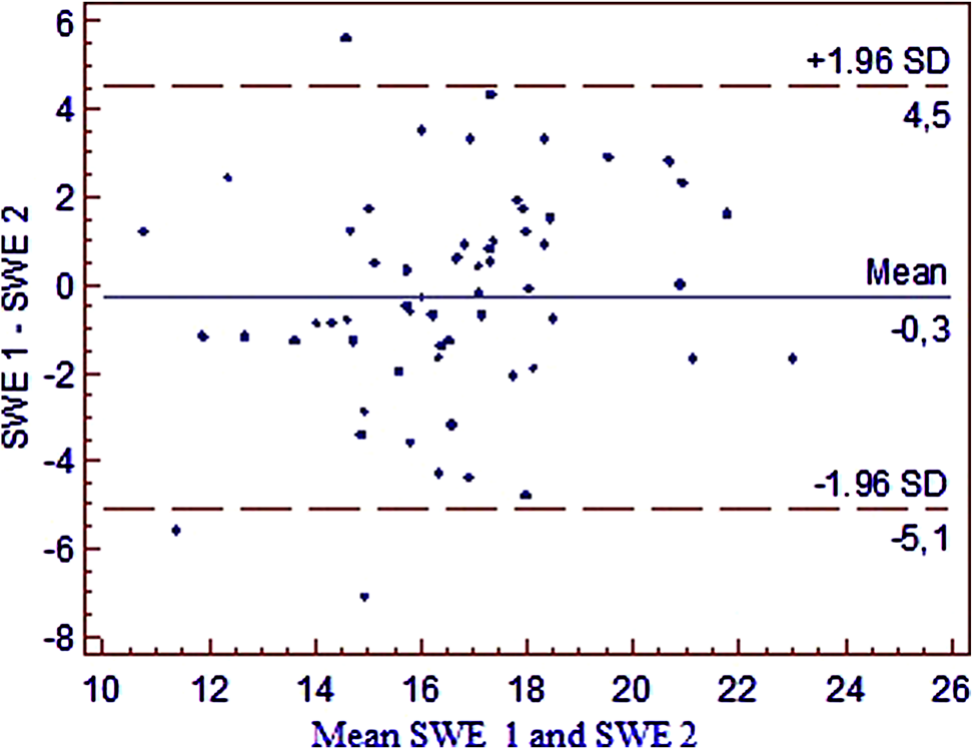
Bland and Altman’s diagram of results of two spleen hardness measurements (SWE 1 and SWE 2)

**Fig. 4:**
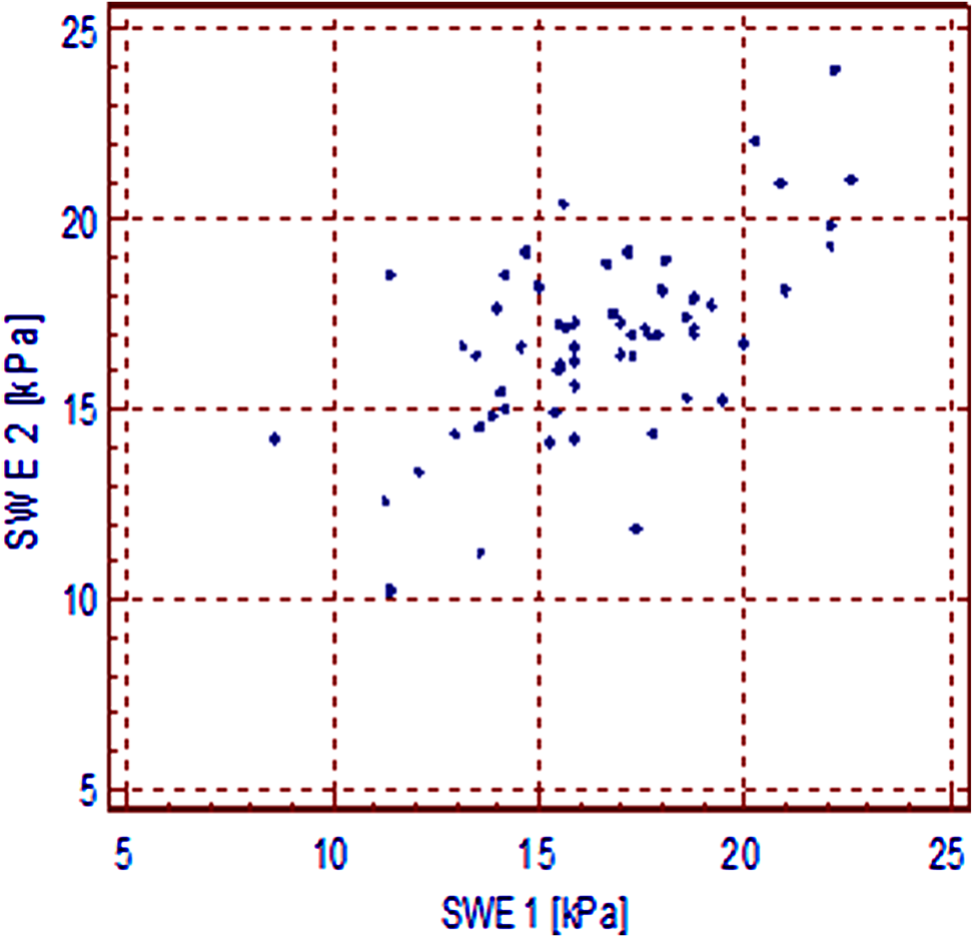
A correlation diagram of SWE 1 vs. SWE 2

Coefficient of Repeatability was calculated as a percentage of the two measurements whose difference exceeded the limit of agreement, rAB = 3/59 = 5.1% (Fig. 3). Correlation coefficient, as shown in Fig. 4, was calculated (as well as 95% confidence interval) between the results of the first and the second measurement: *r* = 0.608 (0.417–0.748), *p* < 0.0001.

To confirm previous calculations, an R&R method was used: %R&R = 23.2 (the value is <30%—this spleen examination system (equipment + examiner) can be conditionally accepted); Repeatability: EV (equipment variation) = 4.37; Reproducibility: AV (appraiser variation) = 1.01; Repeatability and Reproducibility: R&R = 4.48.

The results of this analysis show a high repeatability of the spleen stiffness measurements and indicate that two good quality measurements acquired from the same area of the splenic parenchyma could be sufficient (Figs. 3, 4).

## Discussion

Although manual palpation has been an important element of physical examination for centuries, it was only at the end of the 20th century that brought us the possibility of a graphic and quantitative assessment of tissue stiffness due to elastography. The constant development of this branch of science has led to the creation of many new elastographic techniques. To evaluate the stiffness of the parenchymal organs, usually quantitative techniques, such as TE, ARFI, pSWE, SWE, and elasto-MR, are used. Each of these methods has its advantages and disadvantages [1, 3]. The results of the research based on only one of these techniques show general accuracy. However, the differences in technological and physical basis of these methods as well as various ways of data acquisition do not allow us to extrapolate the results without reflections. The lack of a possibility to visualize the examined area in TE or lack of possibility to regulate the size of ROI in ARFI or in pSWE can be mentioned as examples of potentially significant differences between these methods [3, 10].

Elastography, as a very innovative method, has brought a lot of enthusiasm in the scientific world in the last century. A lot of studies about this method have been published. It might be said that elastography has completely changed the diagnostic process of liver fibrosis. Elastography is also used as a complementary method in diagnosing focal changes in, e.g., the breast(s) and thyroid [3]. The spleen, as a relatively large and available organ for ultrasound examination, seems to be a very promising target for elastography, especially in examination of changes affecting whole parenchyma.

The mean value of spleen stiffness obtained in our research was 16.6 ± 2.5 kPa and it was similar to the result of Leung et al. (17.3 ± 2.6 kPa) [9]. Our study confirmed no correlation between spleen stiffness and sex, age of patients, and spleen size. These results will certainly allow us to simplify the methodology of future spleen stiffness studies.

We strongly believe that our results may be a reference point for evaluating spleen stiffness in research on patients with different illnesses. Our study demonstrated good but not ideal repeatability of measurement results (5.1%). It suggests a necessity to perform at least two measurements of spleen stiffness in an SWE elastography examination.
